# High grade glioma radiation therapy on a high field 1.5 Tesla MR-Linac - workflow and initial experience with daily adapt-to-position (ATP) MR guidance: A first report

**DOI:** 10.3389/fonc.2022.1060098

**Published:** 2022-11-28

**Authors:** Chia-Lin Tseng, Hanbo Chen, James Stewart, Angus Z. Lau, Rachel W. Chan, Liam S. P. Lawrence, Sten Myrehaug, Hany Soliman, Jay Detsky, Mary Jane Lim-Fat, Nir Lipsman, Sunit Das, Chinthaka Heyn, Pejman J. Maralani, Shawn Binda, James Perry, Brian Keller, Greg J. Stanisz, Mark Ruschin, Arjun Sahgal

**Affiliations:** ^1^ Department of Radiation Oncology, Sunnybrook Health Sciences Centre, University of Toronto, Toronto, ON, Canada; ^2^ Physical Sciences Platform, Sunnybrook Research Institute, Toronto, ON, Canada; ^3^ Medical Biophysics, University of Toronto, Toronto, ON, Canada; ^4^ Department of Medicine, Division of Neurology, Sunnybrook Health Sciences Centre, University of Toronto, Toronto, ON, Canada; ^5^ Division of Neurosurgery, Sunnybrook Health Sciences Centre, University of Toronto, Toronto, ON, Canada; ^6^ Division of Neurosurgery, St. Michael’s Hospital, University of Toronto, Toronto, ON, Canada; ^7^ Department of Medical Imaging, Sunnybrook Health Sciences Centre, University of Toronto, Toronto, ON, Canada; ^8^ Department of Neurosurgery and Paediatric Neurosurgery, Medical University, Lublin, Poland

**Keywords:** MR-Linac, glioma radiation, tumor dynamics, functional imaging, adapt-to-position

## Abstract

**Purpose:**

This study reports the workflow and initial clinical experience of high grade glioma (HGG) radiotherapy on the 1.5 T MR-Linac (MRL), with a focus on the temporal variations of the tumor and feasibility of multi-parametric image (mpMRI) acquisition during routine treatment workflow.

**Materials and methods:**

Ten HGG patients treated with radiation within the first year of the MRL’s clinical operation, between October 2019 and August 2020, were identified from a prospective database. Workflow timings were recorded and online adaptive plans were generated using the Adapt-To-Position (ATP) workflow. Temporal variation within the FLAIR hyperintense region (FHR) was assessed by the relative FHR volumes (n = 281 contours) and migration distances (maximum linear displacement of the volume). Research mpMRIs were acquired on the MRL during radiation and changes in selected functional parameters were investigated within the FHR.

**Results:**

All patients completed radiotherapy to a median dose of 60 Gy (range, 54-60 Gy) in 30 fractions (range, 30-33), receiving a total of 287 fractions on the MRL. The mean in-room time per fraction with or without post-beam research imaging was 42.9 minutes (range, 25.0–69.0 minutes) and 37.3 minutes (range, 24.0–51.0 minutes), respectively. Three patients (30%) required re-planning between fractions 9 to 12 due to progression of tumor and/or edema identified on daily MRL imaging. At the 10, 20, and 30-day post-first fraction time points 3, 3, and 4 patients, respectively, had a FHR volume that changed by at least 20% relative to the first fraction. Research mpMRIs were successfully acquired on the MRL. The median apparent diffusion coefficient (ADC) within the FHR and the volumes of FLAIR were significantly correlated when data from all patients and time points were pooled (*R*=0.68, *p*<.001).

**Conclusion:**

We report the first clinical series of HGG patients treated with radiotherapy on the MRL. The ATP workflow and treatment times were clinically acceptable, and daily online MRL imaging triggered adaptive re-planning for selected patients. Acquisition of mpMRIs was feasible on the MRL during routine treatment workflow. Prospective clinical outcomes data is anticipated from the ongoing UNITED phase 2 trial to further refine the role of MR-guided adaptive radiotherapy.

## Introduction

The development of magnetic resonance imaging (MRI)-guided radiotherapy with an integrated high-field strength (1.5 Tesla) MRI-linear accelerator (MR-Linac) enables the daily acquisition of an MRI which allows for on-line soft tissue visualization. Therefore, alignment on the tumor itself is now possible as opposed to relying on a bony surrogate or implanted fiducial and, most importantly, the ability to adapt treatment in real time (1–3). Moreover, a high-field strength MR-Linac permits the acquisition of functional imaging such as diffusion, chemical exchange saturation transfer (CEST), perfusion, and other quantitative MRI (qMRI) biomarkers which introduces the possibility to further individualize treatment (4–10).

At the Sunnybrook Odette Cancer Centre (Toronto, Canada), as a founding member of the Elekta MR-Linac Consortium, our role was to develop the technology primarily for central nervous system tumors (11). Our focus was on the management of intracranial high grade gliomas (HGG) given that following maximal safe resection, radiotherapy with or without concurrent and adjuvant chemo is the standard of care (12–16). The motivation to study this population was based on the lack of any meaningful advances in radiotherapy margin design despite the integration of MRI into radiation planning for almost three decades. In order to determine if personalized margins would be beneficial, predicate work to determine tumor dynamics was undertaken by prospectively imaging patients during a 6-week course of therapy. Stewart et al. reported that inter-fraction volume changes and migration distances are indeed a factor to consider, and challenged the dogma that HGGs are static during a course of chemo-radiotherapy (17). Therefore, when clinical operations of the Unity MR-Linac (Elekta AB, Stockholm, Sweden) began at our institution, we started by treating patients with HGG based on standard margins and contouring practices (18), and evaluated the entire process including our in-house developed workflow, treatment toxicities, and imaging outcomes to inform future directions. In the present study, we report the clinical experience of an initial cohort of 10 HGG patients with an additional focus on the temporal variations of the tumor and the feasibility of acquiring research based multi-parametric images during routine treatment workflow.

## Methods and materials

Ten HGG patients treated on our MR-Linac (MRL) between October 2019 and August 2020 within the first year of the MRL’s clinical operation were identified from a prospective database and retrospectively analyzed. All patients had histologically confirmed WHO Grade 3 or 4 glioma. The study was approved by the institutional ethics review board and all patients provided written consent to be enrolled on the MOMENTUM trial, an international prospective registry designed to facilitate evidence-based implementation of the first MRL and collect outcomes data (19).

### MRL glioma adapt-to-position workflow

**Figure 1:**
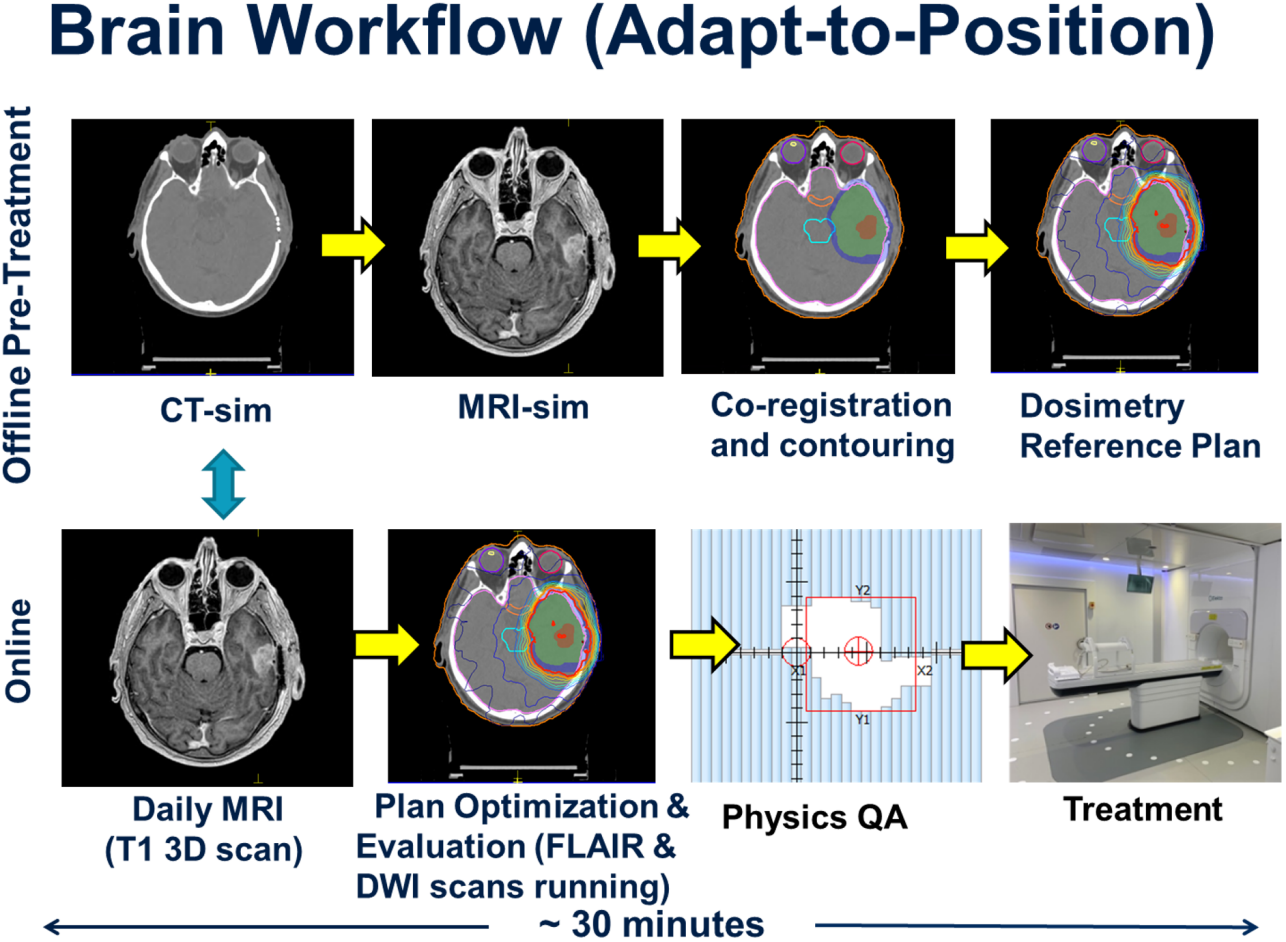
Workflow for both the offline (upper figures) and online (lower figures) series of events in the treatment of the 10 glioma patients. The offline pre-treatment workflow involves initial simulations and treatment planning that leads to a reference plan. The online portion involves a daily MRI scan used to account for patient setup variations and the generation of a new daily online treatment plan as per the adapt-to-position workflow followed by physics quality assurance checks prior to and post treatment. The turquoise arrow indicates the online co-registration between the acquired daily MRI and the reference CT primary data set.

The patient workflow was categorized into offline and online components, and this is depicted in **Figure 1**. The offline (pre-treatment) component began with CT and MRI simulation scans. A CT overlay was placed on the CT simulation couch which mimicked the couch top of the MRL. An MR-safe Orfit (Orfit Industries NV, Belgium) base plate was affixed to the couch overlay and a 3-point mask was used for immobilization. A dummy coil, mimicking the true MRL anterior coil, was used to ensure that there were no collisions between the patient and the coil. The slice thickness for CT scanning was 1 mm. MRI simulation sequences included a post-gadolinium T_1_-weighted 3D sequence, a T_1_-weighted DIXON (fat sat), a T_2_-weighted fluid-attenuated inversion recovery (FLAIR), and a DWI sequence.

The CT and MRI data sets were imported into the Monaco treatment planning system (TPS) to generate a reference plan. The Monaco TPS uses a Monte Carlo dose calculation algorithm that models the effects of the magnetic field (20). The MRI data set was co-registered to the CT data set, and contouring was completed by the treating radiation oncologist. The gross tumor volume (GTV) was defined as the T_1_-weighted gadolinium-enhancing disease for Grade 4 tumors, or FLAIR hyperintense disease for Grade 3 tumors. The clinical target volume (CTV) consisted of a standard 1.5 cm or 1.0 cm expansion beyond the GTV for grade 4 and grade 3 disease, respectively, adjusted for anatomical barriers and routes of spread (18). The PTV expansion was 0.3 cm beyond the CTV. Planning was based on a pre-defined template in the Monaco TPS, which pre-loads the prescription dose, IMRT optimization parameters, beam arrangement (typically 9 beams), dosimetric criteria and reference dose. Treatment plans were calculated using the following parameters: 1% statistical uncertainty, a 3 mm dose grid in all directions, dose calculated to medium using the GPUMCD Monte Carlo algorithm, a minimum segment monitor unit of 2 MU and a minimum segment size setting of 4 cm^2^. A dose reference point was placed in the centre of the PTV, in order to perform an independent dose check during the online planning stage of the patient’s treatment. The reference treatment plan was sent for patient specific quality assurance (QA) measurement using the Arccheck-MR device (Sun Nuclear, Melbourne, Fl) prior to the start of treatment. Backup treatment plans for conventional Linacs were generated in case the MRL was not operational due to unexpected downtime or preventative maintenance.

The online workflow began with acquisition of daily pre-beam non-contrast enhanced MRI sequence (T_1_-weighted 3D volumetric scan), which was co-registered to the pre-treatment reference CT scan. The shift information was used to adjust the patient isocenter location prior to re-optimizing the daily treatment plan. This type of workflow, where the new daily online plan accounts only for rigid translational shifts of the patient, is referred to as the ATP workflow within the Monaco TPS. The Adapt-to-Shape (ATS) workflow, which was not applied to this initial cohort, describes a process by which the organs-at-risk (OAR) and target contours are deformed or re-contoured to reflect the anatomy of the day based on the online MRI, with which the plan is then re-optimized.

All workflow timings were recorded for each patient. During co-registration and planning, FLAIR and DWI MRI sequences were acquired. The new online plan was then reviewed and accepted by the radiation oncologist, or reviewed by the MRL radiation therapist after the first 5 fractions based on a set of pre-defined target coverage objectives and OAR tolerance thresholds, where the treating physician would be paged if tolerances were exceeded. Upon approval of the online plan, an independent dose check was performed using the RadCalc software (Lifeline Software, Tyler, TX), and the dose to a point in the center of the PTV validated. The dose difference between the Monaco TPS and the RadCalc software must be within 4% otherwise an investigation was warranted. For all 10 HGG patients, post treatment patient specific QA measurements were performed using the same criteria as the pre-treatment reference plan measurements.

### FLAIR hyperintense region dynamics assessment

All T2-FLAIR images acquired on the MRL were imported into a research version of the Monaco TPS (Monaco Research v. 5.19.03d; Elekta AB, Stockholm, Sweden). The FLAIR hyperintense region (FHR) was then manually contoured by an attending radiation oncologist (H.C.), and verified by a second radiation oncologist (C.L.T.) at each time point. For each patient, the daily T2-FLAIR image sets from fraction 2 onwards were registered with six degree-of-freedom translational and rotational fusion using a mutual information registration algorithm to the respective 1^st^ fraction T2-FLAIR image set. Following registration, all contours (total n = 281) for each patient were copied to the 1^st^ fraction T2-FLAIR image set, and the RT structure set exported for further analysis. The volumes and migration distances of the contoured FHRs were then computed using the exported structure sets by a custom MATLAB script (MATLAB 2020a; The Mathworks Inc., Natick, Massachusetts). The migration distance was defined as the maximum linear distance in any direction that the contoured FHR volume departs from its respective first fraction volume (17).

### Multi-parametric imaging acquisition and analysis

Research multi-parametric images were acquired on the MRL during radiation treatment, typically post beam-on. The microstructural and functional sequences included DWI, CEST, magnetization transfer (MT), and blood oxygenation level dependent (BOLD) resting-state fMRI. Additionally, variable flip angle and multi-echo sequences were acquired for T_1_ and T_2_ mapping, respectively. All sequences were obtained with whole-brain volumetric coverage, except for the single-slice CEST and three-slice MT scans. Each sequence was obtained at separate treatment fractions, with up to 1 week between repeated measurements for certain sequences. These sequences were not directly used for planning, but were prospectively acquired for research and development. The acquisition protocols are detailed in **Supplementary Material**.

ADC maps were fitted using the mono-exponential model log*S*(*b*)=log*S*_0_-*b·ADC* with b-values of [100,200,400,800] s/mm^2^ with a two-step weighted linear-least squares procedure (21, 22). CEST parameter maps were calculated as described previously (23). The T_1_ map was estimated from the variable flip angle data using the method described in Liberman et al. (24), incorporating uniform weighting of all flip angles and B_1_ inhomogeneity correction applied to the T_1_ map, except that the nominal flip angles were used for fitting. The T_2_ map was estimated from the multi-echo data using a mono-exponential model with linear least-squares fitting of the log-signal versus echo time.

The temporal behaviors of selected functional parameter values within the FHR were investigated. These parameters included ADC, T_1_, T_2_ and CEST asymmetry. For each patient, the medians of the functional parameter values within the FHR contour of the day were computed.

In order to determine the association between ADC values and FHR, the Pearson correlation coefficient between the median ADC and FHR using all treatment fractions was computed per patient. The hypothesis of a non-zero correlation coefficient was tested using a threshold adjusted for multiple comparisons (*α*=0.05/10=0.005). The correlation coefficient was also computed using the median ADC values and FHR for all patients and time points pooled together.

The FLAIR and diffusion-weighted images were co-registered to a reference MRL T_1_-weighted image and the FLAIR-to-reference transformation was then applied to the FLAIR contours. For greater concordance with the b-value range used in previous literature, the ADC maps were re-fitted from the co-registered DWI using b-values of [0,200,400,800] s/mm^2^. A region of low ADC was defined from the maps by taking the largest connected component of the set of voxels within the region of FLAIR hyperintensity having an ADC less than 1.25 μm^2^/ms (25). For those patients who exhibited progressive disease per RANO-HGG (26), the region of recurrent tumor was contoured on the first MRI scan at which progression occurred. The recurrent tumor was defined as the enhancing tumor for Grade 4 patients, and as the FHR for Grade 3 patients. For each patient, the T_1_-weighted image at recurrence was registered to the reference MRL T_1_-weighted image and the same transformation was applied to the contour of recurrent tumor. The overlap between low-ADC regions measured during radiotherapy and the region of recurrence was evaluated.

Overlap was quantified using the Dice score, as well as the sensitivity and positive predictive value (PPV) for the voxel-wise prediction of the region of recurrence by the low-ADC region. These metrics are given by the following equations:


$$\text{Dice}=\frac{2\left|A\cap^{}R\right|}{\left|A\right|+\left|R\right|}$$

$$\text{Sensitivity}=\frac{\left|A\cap^{}R\right|}{\left|R\right|}$$

$$\text{PPV}=\frac{\left|A\cap^{}R\right|}{\left|A\right|}$$

Where *A* is the set of low-ADC voxels, *R* is the set of recurrence region voxels, | | denotes the number of voxels in a set, and *A*∩ *R* is the intersection of *A* and *R*. The sensitivity corresponds to the fraction of the recurrence region contained in the low-ADC region and the PPV with the fraction of the low-ADC region contained in the recurrence region.

### Statistical analysis

Descriptive statistics were used to assess patient demographics, disease characteristics and treatment details. Categorical variables were expressed as counts and proportions, whereas continuous variables such as age and follow-up were expressed as median and range.

Time-to-death was calculated in months from the start date of radiation to date-of-death. Overall survival rates (OS) and progression-free-survival rates (PFS) were obtained using the Kaplan-Meier product-limit method. For OS, patients who were alive at time of analysis or who have become lost to follow-up were censored at their last follow-up date. PFS was defined as the time interval between the start date of radiation until date of disease progression (per RANO-HGG) (26) or death, whichever came first. If neither event had been observed, then the patient was censored at the date of last disease assessment. Statistical analysis was performed using open source statistical software R version 4.0.2 of R for Windows (The R Foundation for Statistical Computing, Vienna, Austria, 2022), and packages prodlim (v2019.11.13) and tableone (v0.12.0).

## Results

All 10 HGG patients completed radiotherapy to a median dose of 60 Gy (range, 54 – 60 Gy) in 30 fractions (range, 30 – 33), receiving a total of 287 fractions on the MRL. Sixteen fractions were delivered with a conventional Linac as a result of either machine downtime or maintenance requirements. The mean in-room time per fraction was 37.3 minutes (range, 24.0 – 51.0 minutes) excluding post-beam research imaging, and 42.9 minutes (range, 25.0 – 69.0 minutes) when post-beam research imaging was performed. All patients met the independent dose check tolerance criterion. Nearly all (90%) received concurrent chemotherapy, and all patients received adjuvant chemotherapy. Patient, tumor, and treatment characteristics are summarized in **Table 1**, and treatment characteristics and clinical outcomes in **Table 2**. The median follow-up time was 25.1 months (range, 3.4 – 31.6 months). The 1-year and 2-year OS rates were 80.0% and 70.0%, respectively. The 1-year and 2-year PFS rates were 60.0% and 50.0%, respectively. No acute grade 3 or higher toxicities were observed.

**Table 1:** Summary of patient, tumor and treatment characteristics.

Characteristics	n = 10
Median age, years (range)	40.0 (29.0 – 69.0)
Gender	
Male Female	3 (30.0%) 7 (70.0%)
WHO Tumor Classification	
GBM, IDH-wild type Astrocytoma, IDH-wild type, grade 3 Astrocytoma, IDH-mutant, grade 4 Astrocytoma, IDH-mutant, grade 3 Oligodendroglioma, IDH-mutant, 1p/19q co-deleted	4 (40.0%) 2 (20.0%) 2 (20.0%) 1 (10.0%) 1 (10.0%)
MGMT Promoter Methylation	
Unmethylated Unknown	4 (40.0%) 6 (60.0%)
Surgery	
Gross Total Resection Subtotal Resection Biopsy	1 (10.0%) 8 (80.0%) 1 (10.0%)
Median no. days from surgery to start of radiation (range)	23.5 (12.0 – 39.0)
Fractionation Scheme	
60 Gy/30 fractions 59.4 Gy/33 fractions 54 Gy/30 fractions	7 (70.0%) 1 (10.0%) 2 (20.0%)
Median % fractions completed on MRL (range)	96.8 (70.0 – 100.0)
Mean in-room time in minutes per fraction^*^ (range)	37.3 (24.0 – 51.0)
Mean in-room time in minutes per fraction including post-beam research imaging (range)	42.9 (25.0 – 69.0)
Radiation Re-plan During Treatment	
Yes No	3 (30.0%) 7 (70.0)
Chemotherapy	
Concurrent TMZ Adjuvant TMZ	9 (90.0%) 10 (100.0%)

GBM, Glioblastoma; MRL, high-field MR-Linac; TMZ, Temozolomide.

*Excluding post-beam research imaging.

**Table 2:** Detailed patient treatment characteristics and clinical outcomes.

Patient	Diagnosis	Age	Surgery	RT Dose (Gy)/No. of Fx	Chemo (TMZ)	Re-plan During RT (Fx No. at transition to re-plan; Reason)	No. of Fx on delivered on MRL	Acute Toxicity	Oncologic Outcomes
1	GBM, IDH wild type	32	STR	60/30	Conc and Adj	Yes (15; tumor and edema progression)	27	Grade 2 headaches, nausea, fatigue	Died
2	GBM, IDH wild type	65	GTR	60/30	Conc and Adj	No	29	None	Died
3	GBM, IDH wild type	62	STR	60/30	Conc and Adj	No	30	None	Died
4	Astrocytoma, IDH mutant, WHO Grade 4	29	STR	60/30	Conc and Adj	Yes (10; edema with midline shift)	21	Grade 2 headaches, nausea, fatigue	Stable Disease
5	Astrocytoma, IDH mutant, WHO Grade 3	36	STR	60/30	Conc and Adj	No	30	None	Stable Disease
6	Oligodendroglioma, IDH mutant, 1p/19q co-deleted, WHO Grade 3	69	STR	59.4/33	Adj	No	32	None	Stable Disease
7	Astrocytoma, IDH wild type, WHO Grade 3	42	Biopsy	60/30	Conc and Adj	No	30	None	Progressed
8	GBM, IDH wild type	57	STR	60/30	Conc and Adj	No	30	None	Stable Disease
9	Astrocytoma, IDH wild type, WHO Grade 3	38	STR	54/30	Conc and Adj	No	29	None	Progressed
10	Astrocytoma, IDH mutant, WHO Grade 4	34	STR	54/30	Conc and Adj	Yes (15; edema)	29	Grade 1 headaches, nausea, fatigue	Stable Disease

RT, Radiation; Fx, Fractions; TMZ, Temozolomide; MRL, high-field MR-Linac; GBM, Glioblastoma; STR, Subtotal resection; GTR, Gross total resection; Conc, Concurrent; Adj, Adjuvant.

**Figure 2:**
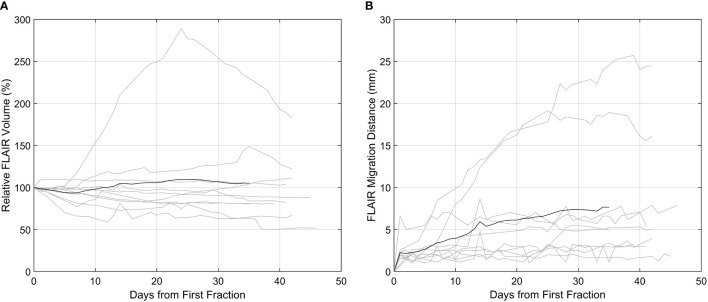
FLAIR hyperintense region (FHR) dynamics as captured on the high-field MR-Linac. In both plots, the patients (n = 10) are delineated by the grey lines, and the black line the mean across all patients. **(A)** T2-FLAIR hyperintense volume relative to the first fraction. **(B)** The migration distance relative to the first fraction.

For all patients, contours from each daily MRI were analyzed for tumor dynamics (n = 281 contours). The FHR dynamics are summarized in **Figure 2**. At the 10, 20, and 30-day post-1^st^ fraction time points 3, 3, and 4 patients, respectively, had a FHR volume that changed by at least 20% relative to the 1^st^ MRL fraction. A relative increase in volume of more than 250% in the FHR was observed in one patient during chemoradiation who required re-planning. In this patient, the change was also associated with a FHR migration distance of more than 25 mm.

Three patients (30%) on temozolomide (TMZ) concurrent with 6 weeks of radiation required re-planning between fractions 9 to 12 due to progression of tumor and/or edema identified on daily MRL imaging. More specifically, Patient 1 was a young woman with a large right frontal GBM, IDH-wild type, who underwent subtotal resection. Progression of edema and associated mass effect was noted on daily MRL FLAIR imaging, and adaptive re-planning performed at fraction 12. The gadolinium-enhanced MRI at the time of re-planning confirmed increased rim enhancement with diffusion restriction concerning for high cellularity. Dexamethasone dosing was increased with improvement in headaches and nausea, and the patient was transitioned to the new adapted plan on the MRL at fraction 15. There was no treatment interruption. Patient 4 was a young man with a right frontal opercular astrocytoma, IDH mutant (non-canonical), WHO grade 4 and underwent subtotal resection. At fraction 9, increased edema with midline shift was observed on the daily MRL FLAIR imaging. The patient’s treatment was held for 2 days while he underwent re-planning and observation on high dose dexamethasone. He was transitioned to the new adapted plan on the MRL at fraction 10 given neurologic stability. Patient 10 was a young man with a left insula, primarily T_2_-weighted hyperintense astrocytoma, IDH mutant, WHO grade 4 who underwent subtotal resection. Interval increased FLAIR hyperintensity surrounding the lesion, with increased midline shift compared to the reference planning images, was observed requiring re-planning at fraction 12. His dexamethasone dosing was adjusted, and he continued radiation with the adapted re-plan started at fraction 15 without any treatment interruption.

**Figure 3:**
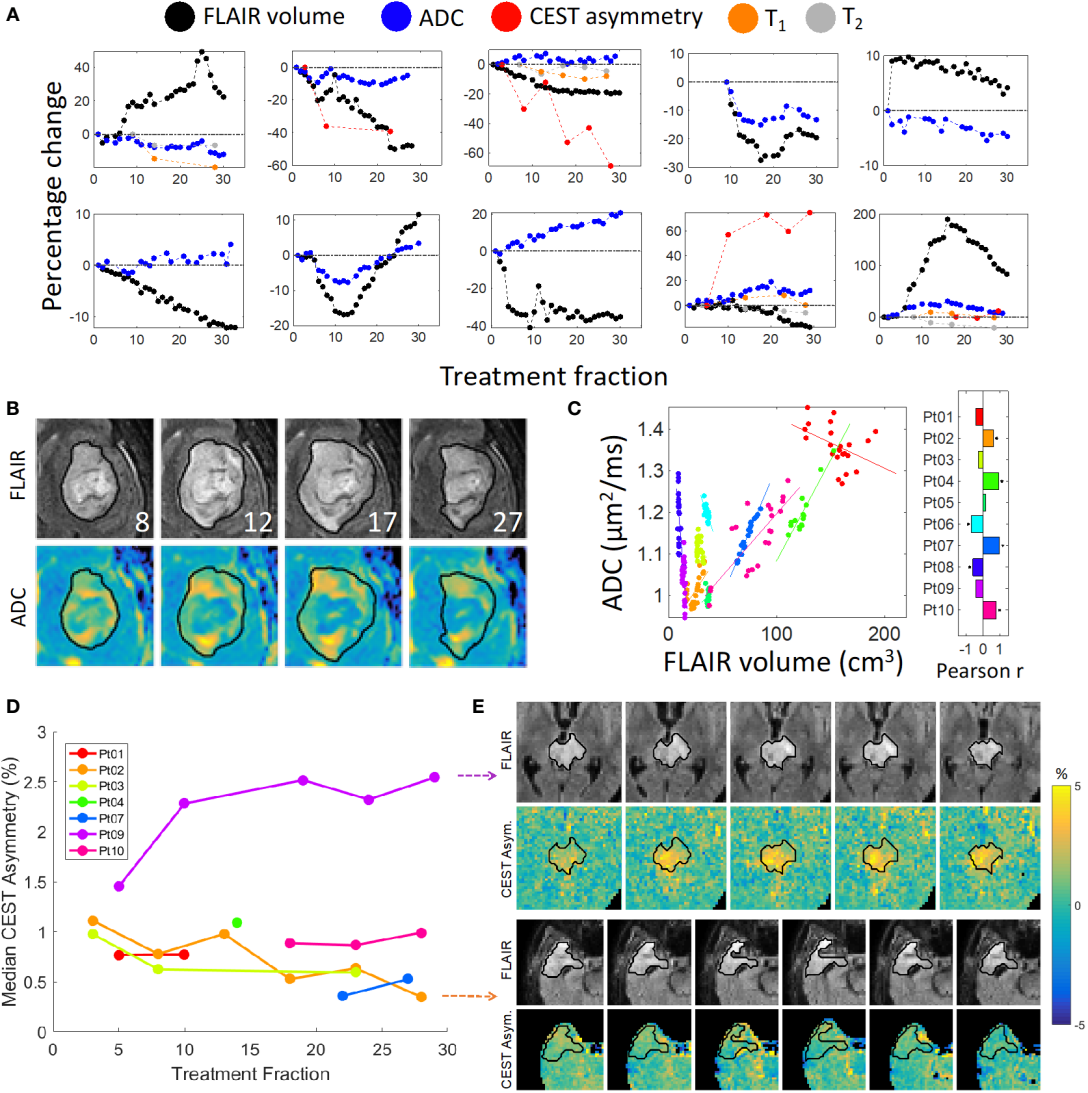
Quantitative imaging and FLAIR contours: **(A)** FLAIR volume and median functional parameter values plotted over time. Zoomed images are shown in **(B)** of the ADC maps for Patient 10 with corresponding FLAIR images with overlaid daily FLAIR contours (in black); the treatment fraction is shown in the lower-right corner of each FLAIR image. A plot of the ADC values vs FLAIR volumes is shown in **(C)**, where each color represents a different patient. The correlation coefficients are shown in the bar plot; an asterisk indicates a statistically significant correlation. **(D)** Median CEST asymmetry with respect to treatment fraction computed over a single slice for the time points with available CEST imaging. Zoomed images are shown for Patient 2 (bottom panel) and Patient 9 (top panel) over time in **(E)** exhibiting decreasing and increasing asymmetry, respectively.

The trends of functional imaging parameters (n = 550 image sequences) during MRL treatment are summarized in **Figure 3**. The correlation between the median ADC value within the FHR and the volume of FHR was statistically significant for 6 of 10 patients (*p*<.05). The magnitude of the correlation coefficient exceeded 0.60 for these 6 patients. The median ADC and FHR volume were also significantly correlated when the data from all patients and time points were pooled (*R*=0.68, *p*<.001).

**Figure 4:**
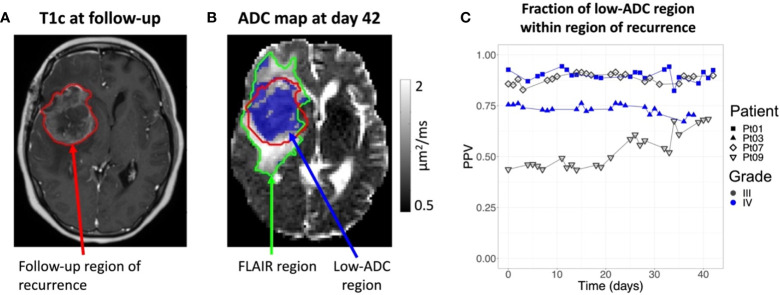
Overlap between intra-treatment low-ADC regions and recurrent tumor: **(A)**: The post-contrast T_1_-weighted image (T1c) of Patient 1 from a diagnostic scanner with the region of recurrence indicated by the red contour. Note that the recurrence region was taken as the enhancing tumor for the Grade 4 patients and the FLAIR hyperintensity for the Grade 3 patients. **(B)**: The ADC map from the MR-Linac for Day 42 from first fraction of radiotherapy, corresponding to the last day of radiation for Patient 1, with the future recurrence region (red contour), FLAIR region (green contour), and low-ADC region (blue colorwash). The low-ADC region is mostly contained in the recurrence region. **(C)**: The positive predictive value (PPV) over time for the voxel-wise prediction of the recurrence region by the low-ADC region. By the end of treatment, most of the low-ADC region is contained within the recurrence region for all patients.

For each of the four patients who exhibited progressive disease during follow-up, the region of recurrence included most of the low-ADC region measured during treatment as illustrated in **Figure 4**. The positive predictive value, reflecting the fraction of the low-ADC region contained in the region of recurrence ranged from 43 – 94% over all patients/time points, and was greater than 68% by the final MRL fraction for all four patients. The sensitivity and Dice scores were lower (1 – 25% and 0.02-0.38, respectively) due to regions of recurrent tumor outside of the low-ADC region.

## Discussion

Daily MRI-guided radiation treatment delivery permits on-line visualization of tumor- and treatment-related temporal changes for HGG patients, which cannot be adequately identified on CT-guided radiation delivery systems. The present study is the first reported clinical series of HGG patients treated with radiotherapy on a high field strength MRL. The ATP workflow and treatment times were clinically acceptable and significant anatomic changes were noted in three of the ten patients which triggered adaptive re-planning. Our observations support the potential for this technology to improve outcomes.

All patients in this study completed radiotherapy as planned with over 96% of fractions delivered on the MRL. Remaining fractions were delivered on conventional Linacs due to machine maintenance or downtime. Despite early concerns of claustrophobia or discomfort as a result of prolonged treatment times on the MRL, no patient discontinued treatment. The tissue-air interface effects have been elucidated in prior planning and *in-vivo* studies suggesting the potential for increased skin and/or air sinus toxicities, but no unexplained toxicities were observed in this cohort (27, 28). Clinically, the treatments were well tolerated with only 2 patients reporting grade 2 acute headaches, nausea and/or fatigue, and 1 patient reporting acute grade 1 symptoms. No acute grade 3 or higher toxicities were observed in this series.

Whether or not tumor- and/or treatment-related temporal changes within the target volume during radiotherapy occur, and if they can lead to geographical misses and compromise patient outcomes, has been an unanswered question. In GBM patients undergoing concurrent chemoradiation over 6 to 6.5 weeks, studies have shown meaningful changes in target dynamics which can occur early in the treatment course (17, 29). Stewart et al. reported a migration distance greater than 5 mm in 46% and 54% of patients for the GTV and CTV, respectively, at fraction 10. Morphologic changes were observed wherein 40% of patients demonstrated a decreased GTV yet with a migration distance of > 5 mm. These data suggest that the majority of target changes occur between time of planning and fraction 10 (17). Bernchou et al. showed similar findings, noting a median maximum distance of > 5 mm between the GTVs at fractions 10, 20, and 30, compared to the original planning GTV (29). These studies provide support for treatment and evaluation of HGG patients with daily MRI guidance with adaptive re-planning as a strategy to account for tumor dynamics. Inter-fraction dynamics may be of critical importance in trials evaluating the safety of CTV margin reduction, as opposed to treatments based on current and historical practices of including 1.5 to 3.0 cm of normal brain tissue in the radiotherapy volume (29).

The present study demonstrated that FHR dynamics can be captured on the MRL, and at least 30% of patients in the study cohort at some point during the treatment course showed a change in FHR volume by 20% or more relative to the 1^st^ fraction. Since gadolinium was not routinely given during the ATP treatment workflow, GTV dynamics could not be assessed in a similar fashion as the prior report by Stewart et al. (17) Importantly, findings noted on daily MRL FLAIR imaging triggered adaptive re-planning in three (30%) patients between fractions 9 to 12, which also led to adjustment in clinical management with respect to steroid dosing as the patients were symptomatic. The relatively early timing of the observed changes was in line with the prior prospective imaging studies. Furthermore, large inter-patient variability in FLAIR hyperintensity dynamics can be observed during the treatment course, and very few patients’ temporal dynamics were closely approximated by that of the mean relative change across the cohort. In one patient (Patient 10), a rapid change in the relative volume of the FHR by nearly 100% was observed between fractions 10 to 20. Hence, the data highlights the benefit of per-fraction daily MR imaging on the MRL in this patient population.

Recent reports from our institution demonstrated feasibility of CEST MRI and DWI acquisition on the MRL for CNS tumors (21, 23), and the current study reporting our initial clinical experience confirms successful acquisition of other multi-parametric image sequences including MT and BOLD resting-state fMRI during routine treatment workflow. ADC parameter changes could be reliably tracked during radiotherapy, and correlation was observed between the median ADC and FHR volumes across all patients and time points. GBM is known to extend beyond the T_1_-weighted contrast-enhancing region on MRI, and outcomes have been correlated with FLAIR abnormalities (30–32). Studies have established an inverse relationship between ADC and glioma cellularity (33–35), therefore, high ADC regions may indicate less cellular tumor within the FHR; however, it is acknowledged that as ADC is not specific to tumor cellularity, elevated ADC may also represent regions of increased edema (36). Therefore, the mixed positive and negative correlation observed between the median ADC and FHR volumes in this series may reflect a combination of tumor cellularity/density and the presence of edema, but a larger sample size is needed to better characterize in future studies. Similarly, association between ADC changes and survival outcomes in HGG have been previously reported (25, 37, 38). This was the first clinical series with daily FLAIR imaging throughout the entire course of radiation as part of the standard treatment workflow. In those patients who developed progressive or recurrent disease, the PPV (corresponding to the fraction of the low-ADC region contained within the region of recurrence) was greater than 68% by the final MRL fraction. Low sensitivity, nonetheless, was observed indicating that tumor recurrence was not confined to only areas of low-ADC. These preliminary findings underscore the importance of further evaluation pending mature clinical outcomes data to better define the role of multi-parametric functional imaging.

The most common pattern of recurrence for HGG post radiation is within or adjacent to the original tumor bed (39, 40). Although the dominant pattern of failure is within the GTV, causes of marginal failures may include positional mis-registration between the volume intended to receive the prescription dose and the actual treated volume, anatomical deformations, and unrecognized tumor progression due to lack of MR guidance at time of treatment delivery. These potential errors could compound over several weeks of treatment. Azoulay et al. reported a 5-fraction course of stereotactic radiation concurrent with TMZ in GBM patients using a 5 mm CTV margin as opposed to the standard 1.5 – 2.0 cm CTV margin approach, which represented a novel approach (41). With a reported marginal failure rate of 11%, the data lent support for possible CTV margin reduction. Patients on this study were treated with standard margins and the intent of the MRL adaptive radiotherapy was to reduce the normal tissue irradiated by compensating for tumor dynamics. An ATP treatment workflow for HGG patients on the MRL enables improved image guidance over that of conventional cone-beam CT-based Linac, but discernment of tumor progression remains challenging in the absence of intravenous contrast. Our ongoing MRL adaptive radiotherapy trial known as UNITED (UNIty-Based MR-Linac Guided AdapTive RadiothErapy for High GraDe Glioma: A phase 2 Trial, NCT04726397), investigates adaptive ATS MRI-guided radiation treatment. The trial is fundamentally based on applying a reduced CTV margin of 5 mm with the option of encompassing adjacent FLAIR signal as a part of the CTV as a personalized approach, and weekly fully re-contoured and re-optimized ATS treatment plans using the on-line gadolinium enhanced T_1_-weighted MRI sequence. Therefore, personalized adapted treatment plans are generated which assures safety of this strategy with the primary endpoint being the patterns of failure.

Our first report of treating HGG patients on the MRL is encouraging with no unexpected grade 3 or higher acute toxicities, and three out of the ten patients were re-planned due to significant changes that would have otherwise resulted in geographical miss. A notable strength of the study is the rigorous follow-up of all patients on the MOMENTUM registry study, with prospective collection of clinical outcomes data including toxicities. Unlike prior studies using a sampling of time points during treatment, temporal variations and functional parameter values from quantitative imaging were successfully evaluated on daily online imaging. Nevertheless, we acknowledge several limitations. The sample size is small and the cohort comprises mixed tumor histological and molecular diagnoses and, therefore, conclusions cannot yet be drawn regarding oncological outcomes. Selection bias could have been present in those patients selected for treatment on the MRL versus those who were ineligible due to contraindications to MRI or other reasons. Finally, significant variability was observed in the temporal trends of the median functional parameter values across the cohort, which can be attributed to the small number of patients and the mixed WHO tumor types and grades. However, we established the feasibility of multi-parametric imaging acquisition on the MRL which will be used in future work to determine if these functional maps can lead to more precise targets for dose escalation or de-escalation.

In conclusion, we report the first clinical series of HGG patients treated with radiotherapy on the Unity 1.5 T high field strength MRL. The ATP workflow and treatment times were clinically acceptable, and daily online MRL imaging triggered adaptive re-planning for selected patients. Acquisition of multi-parametric imaging sequences was feasible on the MRL during routine treatment workflow. ADC parameter changes could be reliably tracked during radiotherapy. Prospective clinical outcomes data based on personalized adapted treatment plans is anticipated from the ongoing UNITED phase 2 trial to further refine the role of MR-guided adaptive radiotherapy on the MRL.

## Data availability statement

The raw data supporting the conclusions of this article will be made available by the authors, without undue reservation.

## Ethics statement

The studies involving human participants were reviewed and approved by the institutional ethics review board at Sunnybrook Health Sciences Centre and all patients provided written consent to be enrolled on the MOMENTUM trial, an international prospective registry designed to facilitate evidence-based implementation of the first MRL and collect outcomes data. The patients/participants provided their written informed consent to participate in this study. Written informed consent was obtained from the individual(s) for the publication of any potentially identifiable images or data included in this article.

## Author contributions

Study concept and design: C-LT, AS. Data collection: C-LT, HC, JS, AL, RC, LL. Data analysis and interpretation: C-LT, HC, JS, AL, RC, LL, AS. Initial draft: C-LT, JS, LL, BK, AS. Revision and approval: All authors contributed to the final writing and revision of the manuscript.

## Conflict of interest

C-LT has received travel accommodations/expenses & honoraria for past educational seminars by Elekta and belongs to the Elekta MR-Linac Research Consortium. JS is employed at Sunnybrook Health Sciences Centre, Toronto, Ontario, Canada. SM has received research support from Novartis AG, honoraria from Novartis AG and Ipsen and travel support from Elekta, none related to this work. SD serves as the Provincial Lead for CNS Oncology at Ontario Health, Cancer Care Ontario; he receives laboratory research support from Alkermes; he serves on the advisory board of the Subcortical Surgery Group and Xpan Medical; he is a speaker for the Congress of Neurological Surgeons. MR is a co‐inventor of and owns associated intellectual property specific to the image‐guidance system on the Gamma Knife Icon, none related to this work. AS has been a consultant for Varian, Elekta Gamma Knife Icon, BrainLAB, Merck, Abbvie, Roche; Vice President of the International Stereotactic Radiosurgery Society ISRS; Co-Chair of the AO Spine Knowledge Forum Tumor; received honorarium for past educational seminars for AstraZeneca, Elekta AB, Varian, BrainLAB, Accuray, Seagen Inc.; research grant with Elekta AB, Varian, Seagen Inc., BrainLAB; and travel accommodations/expenses with Elekta, Varian and BrainLAB. AS also belongs to the Elekta MR Linac Research Consortium and is a Clinical Steering Committee Member, and chairs the Elekta Oligometastases Group and the Elekta Gamma Knife Icon Group.

The remaining authors declare that the research was conducted in the absence of any commercial or financial relationships that could be construed as a potential conflict of interest.

## Publisher’s note

All claims expressed in this article are solely those of the authors and do not necessarily represent those of their affiliated organizations, or those of the publisher, the editors and the reviewers. Any product that may be evaluated in this article, or claim that may be made by its manufacturer, is not guaranteed or endorsed by the publisher.
