# Supplementary material for: High grade glioma radiation therapy on a high field 1.5 Tesla MR-Linac - workflow and initial experience with daily adapt-to-position (ATP) MR guidance: A first report
Source: Front Oncol. 2022 Nov 28;12:1060098. doi: 10.3389/fonc.2022.1060098 (PMC9742425; doi:10.3389/fonc.2022.1060098)
Supplement: Supplementary file 1 [file Table_1.docx]

Supplementary Tables – Acquisition protocols

**Supplementary Table 1 – Diffusion-weighted imaging (DWI) protocol:** Select protocol parameters are listed for the DWI. The number of averages is shown in parentheses after each b-value. Three diffusion encoding directions were acquired with gradient overplus on and the trace image from the scanner was saved. Abbreviations: SS-EPI = single-shot echo planar imaging; SENSE = sensitivity encoding; FE/PE = frequency-encoding/phase-encoding; R = reduction factor.

|  | **DWI** |
| --- | --- |
| **Imaging mode** | SS-EPI (PE direction: anterior - posterior) |
| **Parallel imaging** | SENSE (R=2, anterior - posterior) |
| **TR (ms)** | 4300 |
| **TE (ms)** | 80 |
| **Voxel size (mm^3^)** | 2 × 2.2 × 5.0 |
| **FOV (mm^3^)** | 240 × 240 × 155 |
| **b-values (number of averages)**  **(s/mm^2^)** | 0 (4), 10 (1), 20 (1), 30 (1), 40 (1), 50 (1), 75 (1), 100 (1), 200 (2), 400 (3), 800 (8) |
| **Gradient spacing (**$\boldsymbol{\Delta}$**, ms)** | 39.8 |
| **Gradient duration (**$\boldsymbol{\delta}$**, ms)** | 26.9 |
| **Fat suppression** | SPIR |
| **Readout bandwidth along FE/PE direction (Hz/pixel)** | 1275.0/17.4 |
| **Number of slices** | 31 |
| **Scan time** | 4 min 40 s |

**Supplementary Table 2 – Quantitative magnetization transfer (qMT), T_1_- and T_2_-mapping protocols:** The saturation for the qMT sequence consisted of a series of 10-ms block pulses separated by 2.5-ms gaps with gradient spoilers. Pulse sequence parameters are shown for the qMT, WAter Shift And B1 (WASABI) (used for B_0_ and B_1_ mapping), T_1_ and T_2_ mapping sequences. The total scan duration was 12 min 28 s.


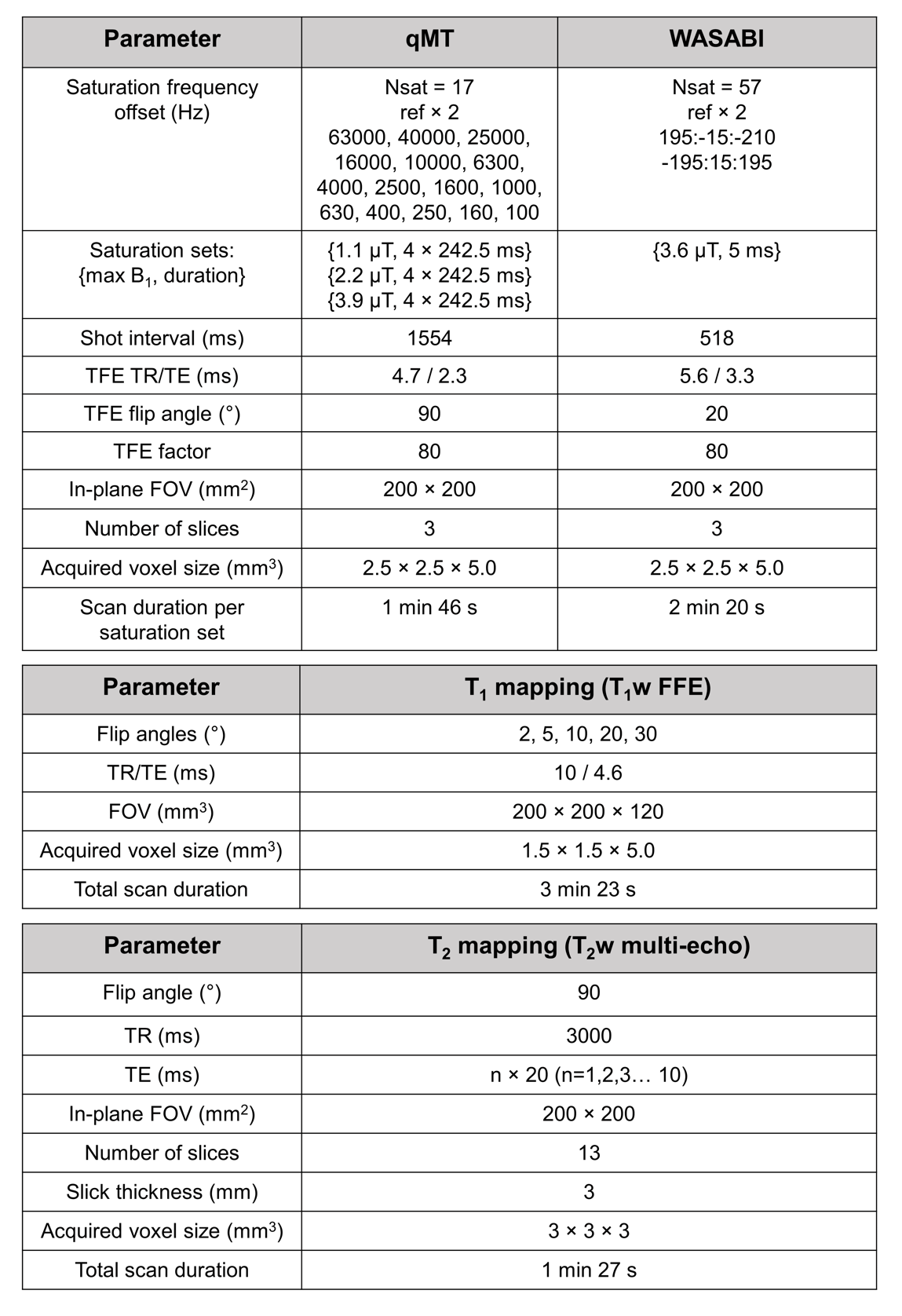


**Supplementary Table 3 – Chemical exchange saturation transfer (CEST) protocols:** Pulse sequence parameters are shown for the CEST and the WAter Shift And B1 (WASABI) sequence. The reference frequency offset (ref) was 100,000 Hz. Sets of 125 rectangular pulses (with duration of 0.5 ms each and gaps of 0.5 ms) were shaped to a Fermi amplitude modulation function, with 9 repetitions of the Fermi-modulated pulses. Scans acquired with 1.5 and 3μT were interpolated to 2.5μT. A subset of scans were acquired directly at 2.5μT to reduce research scan duration.

|  | **CEST** | **WASABI** |
| --- | --- | --- |
| **Saturation frequency offsets (Hz)** | Nsat=202  Sorted across 3 interleaves, each separated by ref×3:  -384:-264, gap=12  -264:-216, gap=6  -216:-168, gap≈3  -168:-12, gap=6  -12:12, gap=3  12:168, gap=6  168:216, gap≈3  216:264, gap=6  264:384, gap=12 | Nsat=57  ref×2,  195:-15:-195  -195:15:195 |
| **Saturation sets: {max B_1_, duration}** | {1.5μT, 0.5ms × 125 × 9 Fermi}  {3.0μT, 0.5ms × 125 × 9 Fermi} | {3.6μT, 5ms} |
| **Shot interval (ms)** | 1500 | 900 |
| **TFE TR/TE (ms)** | 4.6 / 2.3 | 10 / 3.3 |
| **TFE FA (°)** | 80 | 20 |
| **Number of TFE shots; factor** | 1; 80 | 1; 80 |
| **Acquired voxel size (mm^3^)** | 2.5 × 2.5 × 5.0 | 2.5 × 2.5 × 5.0 |
| **Scan duration, per saturation set, per slice** | 5 min 50 s | 1 min 10 s |

**Supplementary Table 4 – BOLD resting-state fMRI protocol:** Select acquisition parameters for the resting-state fMRI protocol. Patients were not given any specific task during acquisition. SS-EPI = single-shot echo planar imaging.

|  | **Resting-state fMRI** |
| --- | --- |
| **Imaging mode** | SS-EPI (PE direction: anterior-posterior) |
| **TR (ms)** | 2600 |
| **TE (ms)** | 40 |
| **Flip angle (°)** | 90 |
| **Voxel size (mm^3^)** | 3.8 × 3.7 × 5.0 |
| **FOV (mm^3^)** | 240 × 240 × 155 |
| **Fat suppression** | SPIR |
| **Readout bandwidth along FE/PE direction (Hz/pixel)** | 2379/22.9 |
| **Number of slices** | 31 |
| **Number of dynamics** | 132 |
| **Scan time** | 5 min 51 s |
